# Healthcare workers preparedness for COVID-19 pandemic in the occupied Palestinian territory: a cross-sectional survey

**DOI:** 10.1186/s12913-021-06804-7

**Published:** 2021-08-03

**Authors:** Osaid Alser, Heba Alghoul, Zahra Alkhateeb, Ayah Hamdan, Loai Albarqouni, Kiran Saini

**Affiliations:** 1 Ministry of Health, Gaza Strip, occupied Palestinian territory and OxPal Medlink, Oxford, UK; 2 Massachusetts General Hospital, Harvard Medical School, 165 Cambridge St, Suite 810, Boston, MA 02114 USA; 3 Faculty of Medicine, Islamic University of Gaza, Gaza, occupied Palestinian territory; 4 Vertex Pharmaceuticals, Boston, USA; 5 Harvard T.H. Chan School of Public Health, Boston, USA; 6 Institute for Evidence-Based Healthcare, Faculty of Health Sciences & Medicine, Bond University, Gold Coast, Australia; 7 Medical Sciences Division, University of Oxford and OxPal Medlink, Oxford, UK

**Keywords:** COVID-19, Coronavirus, PPE, Palestine, Occupied Palestinian territory

## Abstract

**Background:**

The COVID-19 pandemic threatens to overwhelm the capacity of a vulnerable healthcare system in the occupied Palestinian territory (oPt). We aimed to evaluate the availability of personal protective equipment (PPE) and the level of preparedness among HCWs in the oPt.

**Methods:**

A cross-sectional study was conducted using a validated online questionnaire distributed through convenient sampling between March 30, 2020 and April 12, 2020. Outcomes were availability of PPE, healthcare workers (HCWs) preparedness in oPt for COVID-19 pandemic, and regional and hospital differences in oPt in terms of availability of PPE and HCWs preparedness. Descriptive statistics and univariate analysis were used in this study.

**Results:**

Of 138 respondents, only 38 HCWs (27.5%) always had access to facemasks and 15 (10.9%) always had access to isolation gowns. Most HCWs did not find eye protection (*n* = 128, 92.8%), N95 respirators (*n* = 132, 95.7%), and face shields (*n* = 127, 92%) always available. Compared to HCWs in West Bank, those in the Gaza Strip were significantly less likely to have access to alcohol sanitizers (*p* = 0.03) and gloves (*p* < 0.001). On average, governmental hospitals were significantly less likely to have all appropriate PPE than non-governmental institutions (*p* = 0.001). Only 16 (11.6%) surveyed felt confident in dealing with a potential COVID-19 case, 57 (41.3%) having received any COVID-19-related training, and 57 (41.3%) not having a local hospital protocol.

**Conclusion:**

HCWs in oPt appear to be underprepared and severely lacking adequate PPE provision. The lack of PPE provision will exacerbate spread of COVID-19 and deepen the crisis, whilst putting HCWs at risk.

**Supplementary Information:**

The online version contains supplementary material available at 10.1186/s12913-021-06804-7.

## Background

With the ongoing coronavirus disease 2019 (COVID-19) pandemic, several low-to-middle income countries (LMICs) in the Middle East and Africa have reported scarcity of personal protective equipment (PPE) for front line healthcare workers (HCWs) [1, 2]. In the midst of the pandemic, the humanitarian and healthcare crisis in the occupied Palestinian territory (oPt) has exacerbated and the healthcare system further crippled. In the early phase of the pandemic (April 2020), only 268 cases and 2 deaths had been recorded in oPt [3]. However, the number of cases and fatalities progressively increased until reaching its first peak on September 14, 2020 in which the cumulative number of confirmed cases was 6247 and 53 deaths were recorded. This reflects a true ‘first wave’ ripping through the oPt in the interim.

The United Nations Relief and Works Agency (UNRWA) has been unable to support Palestinians’ COVID-19 response needs due to funding cuts and legal restrictions that predate the pandemic [4]. Multiple COVID-19 testing sites serving Palestinians in East Jerusalem have been closed by the Israeli authorities [5]. The West Bank is particularly vulnerable due to checkpoint closures, halting of the transportation of patients to hospitals, and redistribution of clinical supplies. The Gaza Strip is one of the most densely populated places on earth with 2 million inhabitants, mostly refugees, living in 365 sq. km^2^, which would facilitate an accelerated spread of disease during a COVID-19 outbreak [6].

The explicit COVID-19 Response plan of the Ministry of Health in oPt was containment and suppression [7]. A lockdown starting on 22 March, 2020 was anticipated to decimate government revenues and thus the ability to even maintain existing health services; potentially magnifying the disparity between governmental and non-governmental healthcare facilities. Currently the oPt healthcare system is fragmented, with the Ministry of Health, non-governmental organizations (NGOs), the private sector and the UNRWA providing an array of services. Governance and coordination of the overall space is loose [8]. This hinders central PPE procurement and distribution; and tight border controls in the West Bank and blockade of Gaza by Israeli Occupation Authorities additionally impair mobilisation of PPE [8].

We hypothesize that (HCWs) in the oPt are largely underprepared to address COVID-19 in both the West Bank and Gaza Strip. Shortages of PPE pose a serious threat to COVID-19 containment in the oPt. It is also expected that HCWs in the oPt have likely received insufficient training on how to address spread and containment of COVID-19; institutions themselves may not have yet been equipped to draw up or implement preventative or management protocols. To the best of our knowledge, there have been no studies evaluating the preparedness of the HCWs in the oPt to face COVID-19 pandemic. In this study, we aimed to evaluate the availability of PPE and the level of preparedness among the HCWs in the oPt.

## Methods

### Study design, setting and data collection

We conducted a cross-sectional study using an online survey tool. Our survey (*Additional material –* Table 1) was modified from two validated questionnaires; the first was utilized during the H1N1 influenza pandemic [9] and the second one was the Personnel, Infrastructure, Procedures, Equipment and Supplies (PIPES) surgical capacity assessment tool [10]. Our modified questionnaire consisted of 22 questions divided into 3 different sections (respondent and healthcare facility characteristics, availability of PPE, and HCWs preparedness). Availability of PPE and HCWs preparedness were assessed on a 5-point Likert scale. The questionnaire was distributed to HCWs in the oPt through convenient sampling between March 30, 2020 and April 12, 2020. E-mail lists for participants in an educational link (OxPal) and social media (Facebook, Twitter, and LinkedIn) groups of HCWs in oPt were used to disseminate the questionnaire. Participants were required to sign in to limit the number of responses to one per respondent.

**Table 1:** Healthcare workers and their healthcare facility characteristics

Parameter	Value
**Age, median (IQR) - [range]**	28 (24, 35) - [19–57]
**Male, n (%)**	85 (61.6)
**Regional Location, n (%)**	
Gaza Strip	97 (70.3)
West Bank (including East Jerusalem)	41 (29.7)
**Profession, n (%)**	
Medicine	69 (50)
Nursing	35 (25.4)
Physiotherapy	13 (9.4)
Dentistry	6 (4.3)
Other^a^	15 (10.9)
**Level of training, n (%)**	
Student	23 (16.7)
Trainee	23 (16.7)
Non-specialized	69 (50.0)
Specialized	23 (16.7)
**Department / Specialty, n (%)**	
Emergency medicine	20 (14.5)
Surgery (including sub-specialties)	19 (13.8)
Family medicine (primary care)	14 (10.1)
Internal medicine (including sub-specialties)	13 (9.4)
Pediatrics	10 (7.2)
Dentistry	8 (5.8)
Anesthesiology/critical care	7 (5.1)
Obstetrics/Gynecology	7 (5.1)
Radiology	6 (4.3)
Other^a^	13 (9.4)
Not applicable (e.g. students)	21 (15.2)
**Type of healthcare facility (I), n (%)**	
Primary healthcare center/ clinic	43 (31.2)
Secondary hospital	29 (21)
Tertiary (referral) hospital	63 (45.7)
Isolation center	1 (0.7)
Not applicable	2 (1.4)
**Type of healthcare facility (II), n (%)**	
Governmental (Ministry of Health)	98 (71)
Private	26 (18.8)
Non-governmental (NGO) or Mission	13 (9.4)
Not applicable	1 (0.7)

^a^Includes: Optometry, medical secretary, medical laboratory, pharmacy, radiography

### Study outcomes

The primary outcomes assessed were availability of PPE and HCWs preparedness in oPt in the era of COVID-19 pandemic. The secondary outcome was to assess the differences between Gaza Strip and West Bank, and between governmental and non-governmental in oPt in terms of availability of PPE and HCWs preparedness to face the COVID-19 pandemic.

### Statistical analysis

Respondent characteristics were summarized using descriptive statistics. For continuous data, mean and standard deviation (SD) were used to report normally distributed data, while median and interquartile ranges (IQR) were used for non-normally distributed data. For categorical data, results were summarized as counts (n) and percentages (cumulative incidence). Univariate analysis (chi-squared and Fisher’s exact [when *n* < 5] tests) was also used to compare participants’ profession, geographical location, and responses to questions related to the availability of PPE and HCWs preparedness for the COVID-19 pandemic. Likert scale variables were converted from 5-point to binary variables for univariate analysis. For example, ‘often available’, ‘sometimes available’, ‘rarely availably’ and ‘never available’ were grouped together as ‘not always available’ vs ‘always available’. Strongly agree and moderately agree were grouped into ‘agree’ variable, while ‘neutral’, ‘moderately disagree’, and ‘strongly disagree’ were grouped into one variable ‘neutral/disagree’. Missing data were considered missing completely at random, therefore we performed complete case analysis. All statistical analyses were performed using IBM Corp. Released 2019. IBM SPSS Statistics for Windows, Version 26.0. Armonk, NY: IBM Corp.

## Results

Of 140 completed surveys, two were excluded from the study as they were either working outside the oPt or in a non-medical profession.

### HCWs and their healthcare facility characteristics

Of 138 HCWs included in the study, 97 respondents (70.3%) were from Gaza Strip and 41 (29.7%) were from the West Bank. The median (IQR) age was 28 (24–35) years with a range from 19 to 57 years old, and 85 respondents (61.6%) were males. Exactly half of respondents were medical doctors, with approximately 35 (25.4%) in nursing and the remaining quarter in physiotherapy, dentistry, or another health-related profession. 20 (14.5%) of the respondents worked in emergency medicine and 19 (13.8%) in surgery, 14 (10.1%) in primary care and 13 (9.4%) in internal medicine. With regards to place of work, 63 (45.7%) of the respondents worked in a tertiary hospital, 29 (21%) in a secondary facility and 43 (31%) in a primary healthcare centre or clinic. One respondent worked in a COVID-19 isolation centre. 98 (71%) worked in a governmental institution operated by the Ministry of Health, 26 (18.8%) worked in a private hospital and 13 (9.4%) in a non-governmental organisation (NGO) or mission-based place of care (Table 1).

### Availability of PPE and HCWs preparedness in terms of infection control training

Only 67 (48.6%) and 71 (51.4%) of HCWs surveyed indicated that they always had alcohol-based sanitizer and gloves available in their institutions, respectively. Only 38 (27.5%) of respondents indicated that regular face masks were always available when needed, and just over 15 (10.9%) of respondents reported that isolation gowns were always available in their institutions. Over 128 (92.8%), 132 (95.7%), and 127 (92%) of respondents indicated that eye protection, N95 respirators, and face shields were not always available to them at their institutions, respectively. Of HCWs surveyed, 57 (41.3%) indicated that their hospital did not provide a local protocol for the management of COVID-19. Only 57 (41.3%) of respondents had received any COVID-19 related training courses by the time of survey administration. Only 16 (11.6%) of participants agreed with the statement of feeling confident or well-prepared to deal with a potential COVID-19 case (Table 2).

**Table 2:** Availability of PPE and preparedness of healthcare workers in oPt for COVID-19 pandemic

Parameter	n (%)
**Alcohol sanitizer**	
Always available	67 (48.6)
Not always available	71 (51.4)
**Gloves**	
Always available	71 (51.4)
Not always available	67 (48.6)
**Facemask**	
Always available	38 (27.5)
Not always available	100 (72.5)
**N95 respirator**	
Always available	6 (4.3)
Not always available	132 (95.7)
**Isolation gowns**	
Always available	15 (10.9)
Not always available	123 (89.1)
**Eye protection (goggles/glasses)**	
Always available	10 (7.2)
Not always available	128 (92.8)
**Face shields**	
Always available	11 (8.0)
Not always available	127 (92.0)
**All protective measures (above)**	
Always available	15 (10.9)
Not always available	123 (89.1)
**Receipt of any COVID-19 training course (e.g. infection control)**	
Yes	57 (41.3)
No	81 (58.7)
**Hospital has provided local protocol for the management of COVID-19**	
Yes	81 (58.7)
No	57 (41.3)
**I feel confident / well prepared dealing with a potential COVID-19 case**	
Agree	16 (11.6)
Neutral / Disagree	122 (88.4)

### Univariate analysis comparing Gaza strip and West Bank in terms of availability of PPE and HCWs preparedness in terms of infection control training

Compared to the West Bank, respondents from the Gaza Strip reported significantly greater lack of alcohol-based hand sanitizers (*p* = 0.03) and gloves (*p* < 0.001), but no statistically significant differences were observed between regions on other PPE or infection control readiness (Table 3).

**Table 3:** Univariate analysis comparing Gaza Strip and West Bank in terms of healthcare workers preparedness and personal protective equipment (PPE) availability

Parameter	Gaza Strip, n (%)	West Bank, n (%)	***p***-value
	***N*** = 97	***N*** = 41	
**Alcohol sanitizer**			
Always available	41 (42.3)	26 (63.4)	0.026
Not always available	56 (57.7)	15 (36.6)	
**Gloves**			
Always available	40 (41.2)	31 (75.6)	< 0.001
Not always available	57 (58.8)	10 (24.4)	
**Facemask**			
Always available	24 (24.7)	14 (34.1)	0.299
Not always available	73 (75.3)	27 (65.9)	
**N95 respirator**			
Always available	5 (5.2)	1 (2.4)	0.669*
Not always available	92 (94.8)	40 (97.6)	
**Isolation gowns**			
Always available	10 (10.3)	87 (212.2)	0.769
Not always available	5 (5.2)	36 (87.8)	
**Eye protection**			
Always available	8 (8.2)	2 (4.9)	0.723*
Not always available	89 (91.8)	39 (95.1)	
**Face shields**			
Always available	10 (10.3)	1 (2.4)	0.174*
Not always available	87 (89.7)	40 (97.6)	
**All PPE any other measures (above)**			
Always available	12 (12.4)	3 (7.3)	0.552*
Not always available	85 (87.6)	38 (92.7)	
**Receipt of any COVID-19 training course (e.g. infection control)**			
Yes	44 (45.4)	13 (31.7)	0.185
No	53 (54.6)	28 (68.3)	
**Hospital has provided local protocol for the management of COVID-19**			
Yes	58 (59.8)	23 (56.1)	0.709
No	39 (40.2)	18 (43.9)	
**I feel confident / well prepared dealing with a potential COVID-19 case**			
Agree	13 (13.4)	3 (7.3)	< 0.001*
Neutral/disagree	84 (86.6)	38 (92.7)	

*Fisher’s exact test

### Univariate analysis comparing governmental and non-governmental hospitals in terms of availability of PPE and HCWs preparedness in infection control training

On average, governmental hospitals run by the Ministry of Health were also reported by respondents to be significantly lacking in sanitizer, gloves, facemasks, eye protection, and face shields compared to non-governmental institutions (*p* < 0.05) (Table 4).

**Table 4:** Univariate analysis comparing Governmental and non-governmental hospitals in oPt in terms of healthcare workers preparedness and personal protective equipment (PPE) availability

Parameter	Governmental hospitals, n (%)	Non-governmental hospital, n (%)	***p***-value
	***N*** = 98	***N*** = 39	
**Alcohol sanitizer**			
Always available	35 (35.7)	32 (82.1)	< 0.001
Not always available	63 (64.3)	7 (17.9)	
**Gloves**			
Always available	38 (38.8)	32 (82.1)	< 0.001
Not always available	60 (61.2)	7 (17.9)	
**Facemask**			
Always available	15 (15.3)	23 (59.0)	< 0.001
Not always available	83 (84.7)	16 (41.0)	
**N95 respirator**			
Always available	3 (3.1)	3 (7.7)	0.352*
Not always available	95 (96.9)	36 (92.3)	
**Isolation gowns**			
Always available	8 (8.2)	7 (17.9)	0.129
Not always available	90 (91.8)	32 (82.1)	
**Eye protection**			
Always available	2 (2.0)	8 (20.5)	0.001*
Not always available	96 (98.0)	31 (79.5)	
**Face shields**			
Always available	4 (4.1)	7 (17.9)	0.012*
Not always available	94 (95.9)	32 (82.1)	
**All PPE any other measures (above)**			
Always available	5 (5.1)	10 (25.6)	0.001
Not always available	93 (94.9)	29 (74.4)	
**Receipt of any COVID-19 training course (e.g. infection control)**			
Yes	37 (37.8)	19 (48.7)	0.254
No	61 (62.2)	20 (51.3)	
**Hospital has provided local protocol for the management of COVID-19**			
Yes	57 (58.2)	23 (59.0)	1.000
No	41 (41.8)	16 (41.0)	
**I feel confident / well prepared dealing with a potential COVID-19 case**			
Agree	10 (10.2)	6 (15.4)	0.556
Neutral / Disagree	88 (89.8)	33 (84.6)	

*Fisher’s exact test

## Discussion

Our study demonstrates that the availability of PPE in both Gaza and the West Bank is insufficient to support the COVID-19 response needs of the oPt. Alcohol-based hand sanitizers, gloves, face masks, eye protection, isolation gowns, N95 respirators and face shields were reported to be inconsistently available, despite being internationally recommended as critical equipment needed for protecting health care workers from infection [11]. Governmental hospitals, as opposed to non-governmental settings, appear to be particularly lacking in equipment. Lessons from prior outbreaks have underlined the importance of PPE in infection control [12]. Recommendations from the WHO suggest the inadequate supply of infection prevention and control measures must be addressed immediately, with assistance from international partners if necessary [13]. The WHO outlines supplies needed to implement recommended protocols, such as PPE, and denotes them as a key resource for all national authorities currently not producing sufficient volumes themselves. Suggestions for other methods of procurement, conservation and management of PPE have been extensively covered in the literature during the pandemic [14]. Many of these suggestions may not be viable in the geopolitical and economic context in which oPt operates. However, methods such as governmental coordination of all PPE supply, extending or creating new supply through 3D printing all provide viable means of blunting the dearth of PPE in oPt currently [15, 16].

Our study showed that most HCWs surveyed did not receive adequate training on local protocols or measures to address COVID-19 spread. Comparing the preparedness of HCWs in oPt to those around the world is a vital element of the debrief from this pandemic and important in developing strategies to ensure the oPt can face future public health crises. Compared to the literature, a similar study was conducted in Ghana (a low-income country in Africa) in the early phases of the pandemic showed only 7% of HCWs surveyed reported their facilities had enough PPE and 54% had participated in dedicated COVID-19 infection control training [17]. On the other hand, a study assessed HCWs’ preparedness in Saudi Arabia (a high-income country in the Middle East) showed strike differences compared to our study [18]. For example, 95.5% of HCWs surveyed reported receiving COVID-19 infection control training, and most participants showed a “fair” level of overall knowledge about COVID-10 disease. In previous pandemics, clinicians in other countries have been substantially more confident in their clinical ability to manage infected patients than those we surveyed. For example, Chinese ICU HCWs during the 2009 H1N1 pandemic were substantially more confident in their preparedness [19]. This may partly be due to a far greater provision of PPE amongst these workers, permitting greater clinical confidence.

Our study has some important strengths. To our knowledge, this study represents the first attempt to assess the availability of PPE in oPt and the preparedness of HCWs to face the COVID-19 pandemic. We provided a comprehensive evaluation of most PPE described in the literature and used clinically. Participants were well-represented across gender, geographic region, department/specialty, level of training, profession, and type of health care facility.

Potential limitations of this study include a small sample size, which may impact generalizability of our results. The lack of the number of HCWs in Palestine who use the social media platforms we used to disseminate the survey makes it impossible to calculate the response rate. Another weakness of our study was the failure to elicit whether the lack of appropriate PPE was one of the driving factors in reducing HCW confidence in their preparedness. Affirming this association would allow us to assert that attempts to target increasing PPE provision could both protect HCW and improve clinical confidence in managing COVID-19 patients. Potential selection bias arose due to the sampling method; most study participants were recruited from social media posts and emails to the networks of the researchers involved (mainly from Gaza), which may limit the study’s generalisability to the entire oPt population especially those in the West Bank. The sampling methodology could have also contributed to a high representation of physicians, and a lower proportion of female respondents. However, a recent report showed that the majority of healthcare workers in oPt especially physicians and dentists are males ([83%] and [76%], respectively) which could argue against that [20]. However, other studies have demonstrated the viability of social media recruitment and snowball sampling to access difficult to reach populations [21]. Additionally, participants were asked to report on their individual experiences and thus may not be wholly representative of the institutions in which they are employed. The cross-sectional nature of this study is, by definition, unable to take into account any changes in equipment or training preparedness over time and is only representative of the point-in-time data collected. These limitations were acknowledged by the authors during study enrolment - as information was required in a timely manner. This study design allowed the authors to rapidly address the gap in the literature regarding COVID-19’s unique impact on the population in the oPt.

## Conclusions

LMICs are particularly vulnerable to the spread of disease because they often grapple with detrimental resource and financial constraints that existed prior to the spread of pandemic. HCWs in oPt, particularly those in Gaza and in governmental hospitals, appear to lack preparedness to the COVID-19 pandemic and significantly lack adequate PPE provision. The lack of PPE is expected to contribute to the exacerbation of COVID-19 situation and deepen the crisis, whilst putting HCWs at risk. OPt and other LMICs often not only lack proper infrastructure and resources, but also have to navigate restrictions on movement, travel and transportation of essential supplies. The unique geopolitical context of oPt and the structure of its healthcare industry presents additional challenges in mounting a response to a public health crisis. During this global pandemic, procurement of adequate supply of PPE and the development of necessary protocols specific to the unique needs and challenges of the region are urgently needed.

## Supplementary Information

**Additional file 1: Table S1.** Study questionnaire.

## Data Availability

The dataset used and analysed during the current study are available from the corresponding author on reasonable request.

## Abbreviations

COVID-19: Coronavirus disease 19
oPt: Occupied Palestinian territory
HCWs: Healthcare workers
PPE: Personal protective equipment
LMICs: Low-to-middle income countries
WHO: World Health Organization
UNRWA: United Nations Relief and Works Agency
PIEPS: Personnel, Infrastructure, Procedures, Equipment and Supplies
SD: Standard deviation
IQR: Interquartile ranges
NGOs: Non-governmental organizations
