# Supplementary material for: Healthcare workers preparedness for COVID-19 pandemic in the occupied Palestinian territory: a cross-sectional survey
Source: BMC Health Serv Res. 2021 Aug 3;21:766. doi: 10.1186/s12913-021-06804-7 (PMC8329610; doi:10.1186/s12913-021-06804-7)
Supplement: Supplementary file 1 — Additional file 1: Table S1. Study questionnaire. [file 12913_2021_6804_MOESM1_ESM.docx]

| **Supplemental material - Table.1** Healthcare workers preparedness and availability of personal protective equipment in occupied Palestinian territory to face the COVID-19 pandemic | |
| --- | --- |
| **I consent to share my responses for this anonymous survey for research purposes (please tick box)** | |
| **Section I: participant demographics and healthcare facility characteristics** | |
| **Age (years):** |  |
| **Gender:** | a.        Male |
|  | b.       Female |
| **Profession:** | a.        Medicine |
|  | b.       Nursing |
|  | c.        Other (please specify) |
| **Level of training:** | a.        Clinical medical student |
|  | b.       Nursing student |
|  | c.        Intern doctor |
|  | d.       Non-specialized doctor |
|  | e.        Resident |
|  | f.        Registered nurse/staff   nurse |
|  | g.       Nurse practitioner |
|  | h.       Specialist doctor |
|  | i.         Consultant doctor |
|  | j.         Other (please specify) |
| **Department/specialty:** | a.        Internal medicine (including sub-specialties) |
|  | b.       Surgery (including sub- specialties) |
|  | c.        Emergency medicine |
|  | d.       Anesthesiology/critical care |
|  | e.        Family medicine (primary care) |
|  | f.        Pediatrics |
|  | g.       Obstetrics/Gynecology |
|  | h.       Radiology |
|  | i.         Psychiatry |
|  | j.         Non-applicable |
|  | k.       Other (please specify) |
| **Place of work (city/governorate):** | a.        Jerusalem |
|  | b.       Hebron |
|  | c.        Bethlehem |
|  | d.       Ramallah and al-Bireh |
|  | e.        Jenin |
|  | f.        Jericho |
|  | g.       Qalqilya |
|  | h.       Tulkarm |
|  | i.         Tubas |
|  | j.         Salfit |
|  | k.       Nablus |
|  | l.         North Gaza |
|  | m.     Gaza |
|  | n.       Deir al-Balah (includes all cities in middle of   Gaza Strip) |
|  | o.       Khan Younis |
|  | p.       Rafah |
| **Healthcare facility name (in English):** |  |
| **Type of healthcare facility - I** | a.        Primary healthcare center |
| b.       Secondary (district) hospital |  |
|  | c.        Tertiary (referral) hospital |
|  | d.       Other |
| **Type of healthcare facility - II** | a.       Governmental (Ministry of Health) |
|  | b.       Private |
|  | c.        Non-governmental (NGO) or Mission |
|  | d.       Other |
| **Hospital beds (total number)** |  |
| **Section II: Availability of personal protective equipment (PPE) and other protective measures** | |
| **Does your healthcare facility provide/have the following?** |  |
| **Alcohol based hand sanitizer?** | Always available |
|  | Often available |
|  | Sometimes available |
|  | Rarely available |
|  | Never available |
| **Gloves?** | Always available |
|  | Often available |
|  | Sometimes available |
|  | Rarely available |
|  | Never available |
| **Regular facemasks?** | Always available |
|  | Often available |
|  | Sometimes available |
|  | Rarely available |
|  | Never available |
| **N95 respirators?** | Always available |
|  | Often available |
|  | Sometimes available |
|  | Rarely available |
|  | Never available |
| **Isolation gowns?** | Always available |
|  | Often available |
|  | Sometimes available |
|  | Rarely available |
|  | Never available |
| **Eye protection (goggles/glasses)?** | Always available |
|  | Often available |
|  | Sometimes available |
|  | Rarely available |
|  | Never available |
| **Face shields?** | Always available |
|  | Often available |
|  | Sometimes available |
|  | Rarely available |
|  | Never available |
| **All of the above protective measures are readily available in your healthcare facility?** | Always available |
|  | Often available |
|  | Sometimes available |
|  | Rarely available |
|  | Never available |
|  | Never available |
| **Section III: Healthcare workers preparedness for COVID-19 pandemic** | |
| **Have you received any Covid-19 related training course? (e.g. infection control)** | Yes |
|  | No |
| **Does your hospital provide a local protocol for management of COVID-19?** | Yes |
|  | No |
| **I feel confident/well-prepared dealing with a potential COVID-19 case** | Strongly agree |
|  | Moderately agree |
|  | Neutral |
|  | Moderately disagree |
|  | Strongly disagree |
| **Any comments?** | |
|  |  |
